# Diazepam combined with aripiprazole in the treatment of a catatonic stupor patient with venous thrombosis: a case report

**DOI:** 10.3389/fpsyt.2026.1857010

**Published:** 2026-06-05

**Authors:** Haihan Chen, Tingting Lai, Weidong Jin, Honglian Xia, Fengli Sun

**Affiliations:** Department of Psychiatry, Zhejiang Province Mental Health Center, Tongde Hospital of Zhejiang Province, Hangzhou, China

**Keywords:** case report, catatonic, schizophrenia, stupor, venous thrombosis

## Abstract

**Background:**

Catatonic stupor is a potentially life-threatening condition, with key symptoms including mutism, immobility, refusal to eat, and negativism. It may be accompanied by complications such as infection, hyperthermia, and thrombosis. When patients develop concurrent severe physical illnesses, this is very likely to cause delays in diagnosis and treatment, further increasing the risk of clinical deterioration.

**Methods:**

This article reports a 52-year-old patient with schizophrenia presenting with catatonic stupor and failure to establish effective communication. The uniqueness of this case is that the patient was admitted with concurrent pulmonary infection, pulmonary embolism, and lower limb deep venous thrombosis, and modified electroconvulsive therapy (MECT) was associated with substantial clinical risks. The patient was eventually treated with a combination of diazepam and aripiprazole. During treatment, the severity of the patient’s catatonic symptoms was assessed using the Bush-Francis Catatonia Rating Scale (BFCRS).

**Result:**

After combined treatment with diazepam and aripiprazole, the patient’s catatonic symptoms were relieved, and various functions gradually recovered. The BFCRS score reduction rate exceeded 70%, indicating that the expected therapeutic effect was achieved.

**Conclusion:**

As a relatively safe and effective intervention regimen, the combination treatment of diazepam and aripiprazole may be applicable to high-risk catatonic patients with relative contraindications to MECT, such as those complicated with venous thrombosis, providing a new therapeutic option for catatonic stupor.

## 1 Introduction

Catatonic stupor is one of the core clinical manifestations of catatonic syndrome. It is associated with a variety of psychiatric and somatic disorders and is also the most severe clinical subtype of this syndrome (1). Its typical manifestations include mutism, refusal to eat, and immobility, accompanied by increased muscle tone, waxy flexibility, and fixed facial expressions, in severe cases, complete mental and motor inhibition may occur, with a lack of effective response to external stimuli. At the beginning of the last century, Bleuler classified catatonic stupor as a subtype of schizophrenia (2). From a contemporary perspective, catatonic stupor is not unique to patients with schizophrenia; it may also occur in patients with mood disorders, neurological diseases, as well as metabolic and immune system diseases. It is worth noting that the diagnosis of catatonic schizophrenia has been removed from Diagnostic and Statistical Manual of Mental Disorder Fifth Edition(DSM-5)and International Classification of Diseases 11th Revision(ICD-11), and catatonia only exists as an additional specification for schizophrenia (3). However, in psychiatric clinical practice, schizophrenia remains one of the most common primary diagnoses associated with catatonic stupor.

Catatonic stupor is a potentially life−threatening condition that usually requires hospitalization. Severe cases may present with complications such as hyperthermia, malnutrition, pulmonary infection, pulmonary embolism, and even death (3). Therefore, significant challenges exist in its clinical treatment and care. Multiple studies (4–6) have indicated that electroconvulsive therapy (ECT) is an effective treatment for catatonic stupor, especially for patients who fail to respond to medication or are in life−threatening conditions. The study by Federica Luchini (6) showed that ECT achieved a response rate of 80%–100% for all types of catatonic stupor, which was superior to other treatments. Timely ECT intervention should be administered to patients with catatonic stupor, as early intervention can prevent clinical deterioration. Regarding pharmacotherapy, benzodiazepines are among the first−line treatments for adult and adolescent patients, with lorazepam being widely used (7). Compared with the above interventions, the selection of antipsychotics requires careful clinical evaluation. Agents with high D_2_ receptor antagonism and a high risk of extrapyramidal side effects may exacerbate catatonic symptoms such as stupor and negativism, while also increasing the risk of neuroleptic malignant syndrome (NMS) (8, 9). In contrast, most second-generation antipsychotics exert 5-HT_2_A receptor antagonism, which modulates dopamine release in the prefrontal cortex, making them more suitable for the treatment of catatonic schizophrenia (10). Catatonic stupor is closely associated with disrupted GABAergic inhibitory function and dopaminergic imbalance, which provides a theoretical basis for the combined use of diazepam and aripiprazole in clinical intervention.

Over the past few decades, research related to catatonia has received considerable attention from the academic community. This case report describes a patient with catatonia complicated by lower extremity deep venous thrombosis, multiple comorbid risk factors, and therapeutic challenges. We aim to highlight the clinical peculiarity of this case and provide a reference for the clinical treatment of similar patients.

## 2 Case report

### 2.1 Present illness history

The patient was a 52-year-old man who presented with sudden mutism and decreased responsiveness to external stimuli 12 days ago and was diagnosed with schizophrenia. On admission, he was also suffering from pulmonary embolism, lower limb deep venous thrombosis and infection.

The patient’s family provided the following medical history: The patient had good growth and development during childhood. In 1998, after noticing that peers around him were building new houses and getting married, while his family was relatively poor, he gradually developed incoherent speech. He would say “This is my wife” to anyone he saw, and sometimes he talked to himself and spoke to the air. He was diagnosed with schizophrenia. During the course of the illness, he was able to care for himself but did not work. He had been taking clozapine 150 mg/d and risperidone 2 mg/d for a long time.

In November 2025, the patient suddenly developed chest tightness and shortness of breath without an obvious trigger. He was admitted to the emergency department, where pulmonary artery computed tomography angiography (CTA) revealed multiple filling defects in the right upper pulmonary artery branch and the left lower pulmonary artery branch, consistent with pulmonary embolism. Subsequently, the patient developed hyperthermia accompanied by cough and expectoration, which was considered a pulmonary infection. After initial treatment of his physical conditions in the intensive care unit (ICU), the patient was transferred to the psychiatry department for further management of his psychiatric symptoms.

### 2.2 Past medical history

The patient’s previous physical condition was fair. He had a history of hypertension for over 5 years. He had been taking metoprolol tartrate and valsartan amlodipine tablets for long-term treatment, and his blood pressure was generally well controlled. He denied histories of coronary heart disease, diabetes and other related diseases. His vaccination history was up to date. He denied any history of blood transfusion, infectious diseases, surgery or trauma, as well as food and drug allergies.

### 2.3 Physical and mental examination

The body temperature was 36.8°C, blood pressure was 110/84mmHg, and heart rate was 115 bpm. The patient was restrained to bed, with a nasogastric tube and urinary catheter in place. Mental state assessment: The patient exhibited a dull gaze, and a valid mental state evaluation could not be completed.

### 2.4 Laboratory examinations

Lower extremity arteriovenous ultrasound: left lower leg intermuscular vein thrombosis.

### 2.5 Clinical treatment and outcomes

When the patient was transferred from the ICU to our department, his limbs were restrained to the bed. He had an indwelling nasogastric tube for enteral feeding and a urinary catheter, with continuous electrocardiogram monitoring. He remained in a passive state, requiring full assistance and supervision for feeding and medication administration. No clinical signs of aspiration were noted. In terms of treatment, clozapine was maintained at a stable dose for psychiatric symptom control, whereas risperidone was gradually tapered and discontinued. Considering that the patient had a pulmonary infection and an elevated high-sensitivity CRP level, cefoperazone sodium and sulbactam sodium was administered for anti-infective therapy. Concurrently, low-molecular-weight heparin was used to treat lower limb deep venous thrombosis.

**Figure 1:**
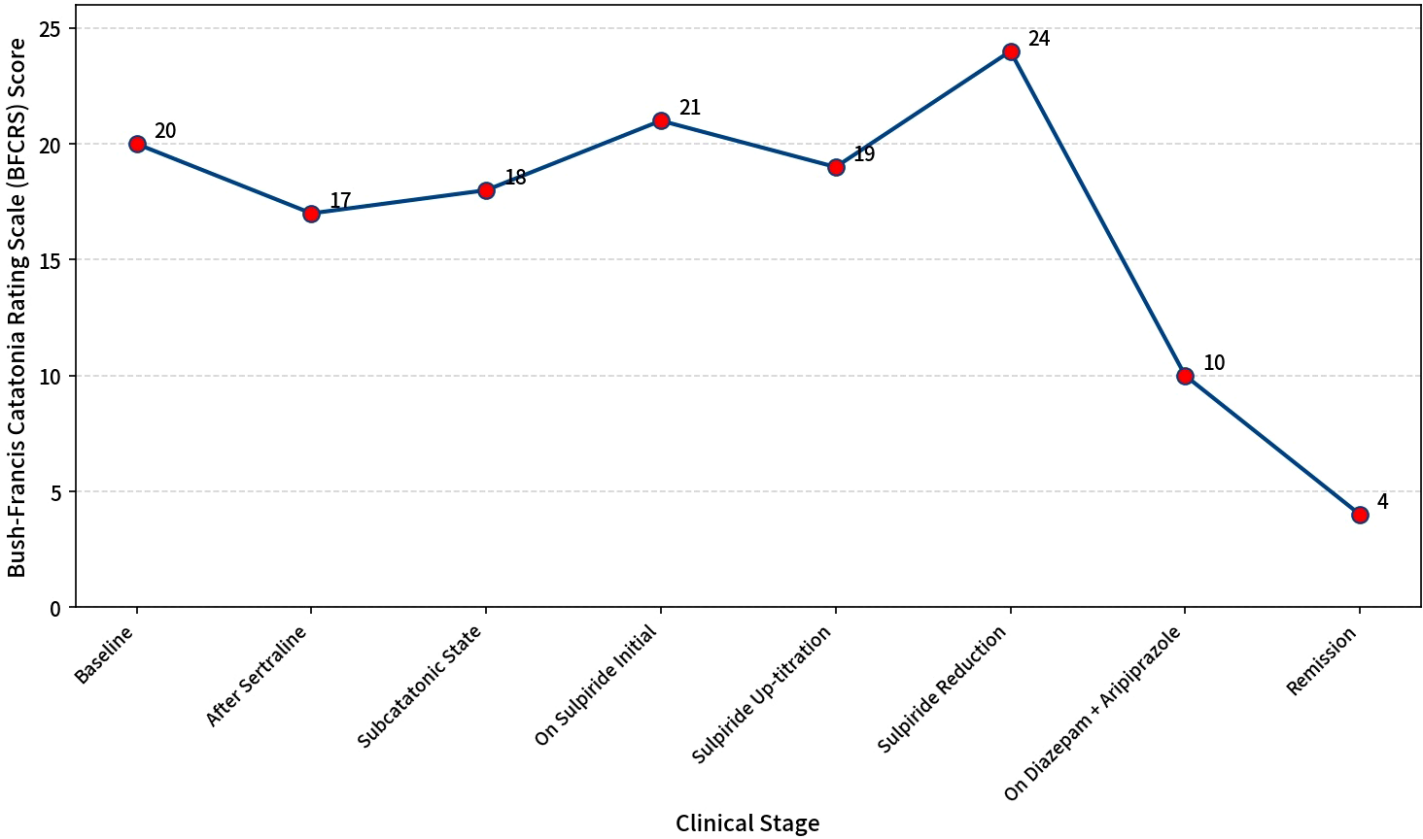
Trend of BFCRS scores during pharmacological treatment in the patient with catatonic stupor.

At the initial stage of treatment, the patient was uncooperative during contact, and effective communication could not be established. His eye contact was poor, but he occasionally responded to the doctor’s questions by nodding, shaking his head, or making corresponding movements. The patient was assessed with the Bush-Francis Catatonia Rating Scale (BFCRS), which comprises 23 catatonic items including excitement, mutism, posturing/catalepsy, rigidity, and negativism. Each item is scored on a 0–3 scale: 0 = absent, 1 = occasional, 2 = frequent, 3 = persistent or severe. The baseline BFCRS score was 20. Through brief communication, it was learned that the patient had previously presented with low mood and excessive worry. In treatment, the dosage of risperidone was gradually reduced, and sertraline at 50–150 mg/d was administered to alleviate his symptoms. With medication adjustment, the patient became more cooperative in communication, with faster responses and more coherent thinking. However, he still required supervision from a caregiver for eating and medication, and the effect was not sustained. Soon afterwards, the patient again became immobile and mute, with a fixed open stare and absent eye contact, and showed negativism. He needed prompting from a caregiver for eating and toileting. At times, he experienced profuse sweating and sustained flexion of both upper extremities, yet appeared to understand part of the staff’s speech, indicating a subcatatonic state. The BFCRS score was 18. Based on the patient’s onset pattern, clinical manifestations, and therapeutic response to sertraline, and we again inquired with the patient’s family, stating that at the beginning of this onset, there was no obvious sign of depression. We systematically ruled out the possibility that the patient’s catatonic stupor was mainly induced by depressive mood. The dosage of sertraline was gradually tapered, and treatment was switched to sulpiride at 100–400 mg/d to improve his condition. At this time, the patient remained passive, mute and immobile, unable to eat, drink, or pass stool independently. There was no verbal communication; he was in a catatonic stupor. The BFCRS score was 21. With gradual up−titration of sulpiride, the patient’s catatonic state partially improved. Although he still lacked eye contact, he could engage in brief conversation with the doctor. The caregiver reported that the patient could walk a few steps passively but required pushing or support to move forward. Overall, communication and interaction improved compared with before, but basic daily activities still required assistance from a caregiver. Although the patient’s catatonic symptoms improved at the initial stage of sulpiride treatment, gradual dose escalation subsequently induced dysautonomia, accompanied by profuse sweating, paroxysmal muscle tension and paroxysmal spasms; each episode lasted 1–2 hours. Ambulatory electroencephalography showed no obvious abnormalities. Since a correlation with the increased dosage of sulpiride could not be ruled out, the dose of sulpiride was gradually reduced. During the dose reduction, the patient’s symptoms relapsed: he kept his eyes closed, was mute and immobile, showed no response, did not answer the doctor’s questions, and had no obvious reaction even to the supraorbital pressure test. Reassessment of catatonia severity showed a BFCRS score of 24, indicating severe catatonia. We considered performing modified electroconvulsive therapy (MECT) for the patient. However, given his admission history of pulmonary embolism and lower limb deep venous thrombosis, the patient was considered at extremely high risk of thrombus dislodgement leading to fatal pulmonary embolism during MECT. After careful discussion and analysis, this plan was abandoned. As an alternative regimen for catatonic stupor, we initiated a combined treatment of diazepam plus aripiprazole. After treatment with diazepam 10 mg/d and aripiprazole 5–20 mg/d, the patient’s catatonic symptoms gradually improved: he was able to open his eyes, have brief conversations with staff, eat and toilet independently under caregiver supervision. With ongoing treatment, his condition continued to improve, and the BFCRS score decreased to 4. One week later, the patient was able to eat, toilet, and walk in the ward corridor independently under supervision. All functions gradually recovered. The BFCRS reduction rate exceeded 70%, indicating the desired therapeutic effect was achieved. The dynamic changes in BFCRS scores during treatment are shown in **Figure 1**.

## 3 Discussion

Catatonic stupor in patients with schizophrenia markedly increases the risk of complications and mortality and may induce overactivation of both the sympathetic and parasympathetic nervous systems. Overactivity of the sympathetic nervous system, dehydration, and prolonged immobility are common features of catatonia (11). Excessive sympathetic activation is characterized by blood pressure lability, tachycardia, tachypnea, and hyperhidrosis (12). The common type of catatonic stupor in schizophrenia usually does not involve severe somatic disorders. The uniqueness of the case reported in this article is that the patient had been diagnosed with schizophrenia for more than 20 years, and upon admission, he had a pulmonary infection, pulmonary embolism, and lower limb deep venous thrombosis of the lower extremities. Shortly after admission, the patient developed catatonic symptoms, including mutism, immobility, inability to eat independently, and the typical sign of catatonia— psychological pillow (13). It is unclear whether the catatonia stemmed from the underlying schizophrenia, the physical comorbidities, or a combination of both. Given the patient’s multiple comorbid physical conditions, including dysautonomia and deep vein thrombosis, MECT was associated with substantial clinical risks. Eventually, after treatment with diazepam combined with aripiprazole, the patient’s catatonic state was gradually relieved.

Catatonic stupor is considered to be closely related to dysfunction of the motor system, especially hyperactivity of the supplementary motor area (SMA) and pre-supplementary motor area (pre-SMA). These regions are crucial for motor control and are modulated by γ-aminobutyric acid (GABA) (14). Benzodiazepines, as positive allosteric modulators of GABA-A receptors, represent one of the main therapeutic strategies for catatonia (15). As a representative benzodiazepine, diazepam enhances GABA-mediated neurotransmission by acting on GABA-A receptors in the brain, strengthens the inhibitory effects of GABA on neurons, and thus exerts clinical effects including sedation, anxiolysis, muscle relaxation, and antispasmodic action, thereby effectively relieves muscle rigidity. Notably, clinical studies (16, 17) have indicated that two patients with GABA-A receptor antibody-related diseases developed catatonic stupor, further supporting the role of the GABAergic system in this pathological process. Recent study (14) by Hidetaka Tamune using electroencephalography in catatonic patients also demonstrated that benzodiazepines can significantly increase alpha and beta wave power across the brain, suggesting that these agents may help restore attention, consciousness, and long-range neural synchrony.

As one of the atypical antipsychotic drugs, aripiprazole exerts unique pharmacological effects. It does not completely block dopamine D2 receptors but instead acts as a partial agonist on these receptors (18), and it has relatively few side effects, rarely causing movement disorders or metabolic syndrome (19). This property reduces the risk of excessive sedation and helps improve negative symptoms such as emotional apathy and social withdrawal, as well as cognitive function, thereby creating favorable conditions for the recovery of the patient’s social functions (19). Some studies suggest that catatonia stupor is not only associated with GABA neurotransmission disorders but also correlates with dopaminergic dysfunction (especially involving D2 receptors) in the cortico-striatal circuit, and there may be a state of relatively low dopamine function (20). Therefore, as a partial dopamine agonist, aripiprazole works through partial agonistic effects to increase dopamine activity in regions with lower dopamine activity, such as the mesolimbic dopamine system in patients with schizophrenia. It may be beneficial for schizophrenia patients with schizophrenia complicated by catatonic stupor (21, 22). Previous studies (23, 24) have also supported the use of aripiprazole in the treatment of catatonic stupor. Moreover, aripiprazole has a weak sedative effect, good tolerability, and overall higher safety profile.

The limitation of this case is that in the initial treatment phase, we were concerned that early benzodiazepine administration might interfere with the etiological identification of catatonia, so benzodiazepines were not administered. We trialed alternative interventions including antidepressants and sulpiride; however, none achieved sustained relief of the patient’ s catatonic symptoms. During gradual up-titration of sulpiride, the patient also developed paroxysmal muscle tension and spasms. This may be attributed to the fact that low-dose sulpiride elevates dopamine levels, contributing to modest symptom relief. By contrast, enhanced dopamine receptor blockade at higher doses may increase the risk of severe clinical deterioration.

In this case, after treatment with diazepam combined with aripiprazole, the patient’s catatonic stupor was successfully relieved and the patient achieved clinical recovery. This result indicates that this combined regimen is effective for catatonic stupor, even for patients with recurrent episodes and severe physical comorbidities. Compared with MECT, treatment with diazepam plus aripiprazole has higher safety and fewer contraindications. It is particularly suitable for patients with relative high-risk conditions to MECT and can serve as a safe and effective alternative therapy for this high-risk population.

## Data Availability

The original contributions presented in the study are included in the article/supplementary material. Further inquiries can be directed to the corresponding author.

## References

[1] Walther S Stegmayer K Wilson JE Heckers S . Structure and neural mechanisms of catatonia. Lancet Psychiatry. (2019) 6:610–9. doi: 10.1016/s2215-0366(18)30474-7 31196794 PMC6790975

[2] Wilson JE Niu K Nicolson SE Levine SZ Heckers S . The diagnostic criteria and structure of catatonia. Schizophr Res. (2015) 164:256–62. doi: 10.1016/j.schres.2014.12.036 25595653

[3] Appiani FJ Castro GS . Catatonia is not schizophrenia and it is treatable. Schizophr Res. (2018) 200:112–6. doi: 10.1016/j.schres.2017.05.030 28610803

[4] Dhossche DM Withane N . Electroconvulsive therapy for catatonia in children and adolescents. Child Adol Psych Cl. (2019) 28:111–20. doi: 10.1016/j.chc.2018.07.007 30389071

[5] Gil-Badenes J Giménez-Palomo A Duque L Pujol-Fontrodona G Martínez-Amorós E Bioque M . Maintenance electroconvulsive therapy in catatonia: Clinical profiles from a case series. J ECT. (2024) 40:173–6. doi: 10.1097/YCT.0000000000001002 38412188

[6] Luchini F Medda P Mariani MG Mauri M Toni C Perugi G . Electroconvulsive therapy in catatonic patients: Efficacy and predictors of response. World J Psychiatry. (2015) 5:182–92. doi: 10.5498/wjp.v5.i2.182 26110120 PMC4473490

[7] Benarous X Raffin M Ferrafiat V Consoli A Cohen D . Catatonia in children and adolescents: New perspectives. Schizophr Res. (2018) 200:56–67. doi: 10.1016/j.schres.2017.07.028 28754582

[8] Sienaert P Dhossche DM Vancampfort D De Hert M Gazdag G . A clinical review of the treatment of catatonia. Front Psychiatry. (2014) 5:181. doi: 10.3389/fpsyt.2014.00181 25538636 PMC4260674

[9] Lee JWY . Neuroleptic-induced catatonia. J Clin Psy. (2010) 30:3–10. doi: 10.1097/jcp.0b013e3181c9bfe6 20075641

[10] Daniels J . Catatonia: Clinical aspects and neurobiological correlates. J Neuropsy Clin Neurosci. (2009) 21:371–80. doi: 10.1176/jnp.2009.21.4.371 19996245

[11] Funayama M Takata T Koreki A Ogino S Mimura M . Catatonic stupor in schizophrenic disorders and subsequent medical complications and mortality. Psy Med. (2018) 80:370–6. doi: 10.1097/psy.0000000000000574 29521882 PMC5959200

[12] Bush G Fink M Petrides G Dowling F Francis A . Catatonia. I. Rating scale and standardized examination. Acta Psychiatr Scand. (1996) 93:129–36. doi: 10.1111/j.1600-0447.1996.tb09814.x 8686483

[13] Shukla R Ahsan M Pal A Shaan F . Unraveling the enigma of 'Psychological Pillow': A unique catatonic phenomenon. Ind Psychiatry J. (2024) 33:S284–6. doi: 10.4103/ipj.ipj_319_23 PMC11553635 39534137

[14] Tamune H Tsukioka Y Sakuma S Taira D Takaoka Y Tamura N . EEG and video documentation of benzodiazepine challenge in catatonic stupor: A case report. Neuropsychopharmacol Rep. (2024) 44:468–73. doi: 10.1002/npr2.12427 38453164 PMC11144595

[15] Rogers JP Pollak TA Blackman G David AS . Catatonia and the immune system: A review. Lancet Psychiatry. (2019) 6:620–30. doi: 10.1016/s2215-0366(19)30190-7 31196793 PMC7185541

[16] Nikolaus M Knierim E Meisel C Kreye J Prüss H Schnabel D . Severe GABA(A) receptor encephalitis without seizures: A paediatric case successfully treated with early immunomodulation. Eur J Paediatric Neurol EJPN Off J Eur Paediatric Neurol Soc. (2018) 22:558–62. doi: 10.1016/j.ejpn.2018.01.002 29396172

[17] Pettingill P Kramer HB Coebergh JA Pettingill R Maxwell S Nibber A . Antibodies to GABAA receptor α1 and γ2 subunits: Clinical and serologic characterization. Neurology. (2015) 84:1233–41. doi: 10.1212/WNL.0000000000001326 PMC4366091 25636713

[18] Ueda T Ugawa S Ishida Y Shimada S . Geissoschizine methyl ether has third-generation antipsychotic-like actions at the dopamine and serotonin receptors. Eur J Pharmacol. (2011) 671:79–86. doi: 10.1016/j.ejphar.2011.09.007 21951966

[19] Tuplin EW Holahan MR . Aripiprazole, a drug that displays partial agonism and functional selectivity. Curr Neuropharmacol. (2017) 15:1192–207. doi: 10.2174/1570159x15666170413115754 28412910 PMC5725548

[20] Northoff G . Catatonia and neuroleptic Malignant syndrome: Psychopathology and pathophysiology. J Neural Transm Vienna Austria 1996. (2002) 109:1453–67. doi: 10.1007/s00702-002-0762-z 12486486

[21] Stahl SM . Dopamine system stabilizers, aripiprazole, and the next generation of antipsychotics, part 2: Illustrating their mechanism of action. J Clin Psychiatry. (2001) 62:923–4. doi: 10.4088/jcp.v62n1201 11780870

[22] Stahl SM . Dopamine system stabilizers, aripiprazole, and the next generation of antipsychotics, part 1, "Goldilocks" actions at dopamine receptors. J Clin Psychiatry. (2001) 62:841–2. doi: 10.4088/jcp.v62n1101 11775041

[23] Kirino E . Prolonged catatonic stupor successfully treated with aripiprazole in an adolescent male with schizophrenia: A case report. Clin Schizophr Related Psy. (2010) 4:185–8. doi: 10.3371/csrp.4.3.5 20880829

[24] Cai ZG . Comparison analysis of the therapeutic effects of diazepam and sulpiride on catatonic state. Med J Chin People's Health. (2012) 24:948. doi: 10.3969/j.issn.1672-0369.2012.08.034

